# Body weight has no impact on self-esteem of minority children living in inner city, low-income neighborhoods: a cross-sectional study

**DOI:** 10.1186/1471-2431-14-19

**Published:** 2014-01-24

**Authors:** William W Wong, Carmen Mikhail, Christina L Ortiz, Debra Lathan, Louis A Moore, Karen L Konzelmann, E O’Brian Smith

**Affiliations:** 1 Department of Pediatrics, Baylor College of Medicine and USDA/ARS Children’s Nutrition Research Center, Houston, TX, USA; 2 Texas Children’s Hospital and Department of Pediatrics, Baylor College of Medicine, Houston, TX, USA; 3 Houston Parks and Recreation Department, City of Houston, Houston, TX, USA

**Keywords:** Self-esteem, Children, Minority, Obesity

## Abstract

**Background:**

The relationship between body weight and self-esteem among underserved minority children is not well documented.

**Methods:**

We measured the self-esteem profile using the Self-Perception Profile for Children among 910 minority children at 17 Houston community centers.

**Results:**

Weight status had no effect on any of the self-esteem scores among the minority children (P ≥ 0.21). Black children had higher scholastic competence than Hispanic children (P = 0.05). Social acceptance was not affected by age, gender, and race/ethnicity (P ≥ 0.13). Significant age x gender (P = 0.006) and race x gender (P = 0.005) interactions were detected on athletic competence. The younger boys had higher athletic competence than the younger and older girls (P ≤ 0.01). The older boys had higher athletic competence than the older girls (P = 0.008) but their scores were not different from those of the younger girls (P = 0.07). Within each race/ethnicity group, boys had higher athletic competence than girls (P ≤ 0.03). Black boys had higher athletic competence than Hispanic girls (P = 0.007) but their scores were not different from those of the Hispanic boys (P = 0.08). Age and gender had no effect on physical appearance but black children had higher scores than Hispanic children (P = 0.05). Behavioral conduct was not affected by age, gender, or race/ethnicity (P ≥ 0.11). There was an age x gender interaction on global self-worth (P = 0.02) with boys having similar scores regardless of ages (P = 0.40) or ethnicity (P = 0.98). However, boys from both age groups had higher global self-worth than the older girls (P ≤ 0.04) but their scores were not different from those of the younger girls (P ≥ 0.07).

**Conclusions:**

For the first time, we documented that being normal weight did not necessarily guarantee positive self-esteem among minority children. Their self-esteem scores were similar to those found among children who were diagnosed with obesity and obesity-related co-morbidities and lower than those reported among normal-weight white children. Therefore, activities to promote self-esteem are important when working with underserved minority children in order to promote a healthy lifestyle.

## Background

Childhood obesity is reaching epidemic proportion in the United States, particularly among minority children [1]. The 2008 Census (http://www.cdc.gov/nchs/data/hestat/obesity_child_07_08/obesity_child_07_08.htm), documented over 5.4 million obese and 4.6 million overweight children and adolescents. A recent Thomson Medstat Research Brief (http://www.healthykidshealthycommunities.org/sites/default/files/FactsAboutChildhoodObesity_2009-06.pdf) indicated that the annual national cost of childhood obesity in the United States is estimated at approximately $11 billion for children with private insurance and $3 billion for those with Medicaid. Alarmingly, healthcare costs associated with obesity in the United States have been documented to outrank the healthcare costs of both smoking and drinking [2]. The U.S. Surgeon General estimated that the annual medical cost associated with obesity is upwards of $117 billion [3]. Using a multiyear data file from the National Hospital Discharge Survey between 1979 and 1999 for youth aged 6–17 years in the United States, obesity-related annual hospital costs were found to increase from $35 million during 1979–1981 to $127 million during 1997–1999 [4]. The most alarming findings from the report were the 2-fold increase in type 2 diabetes, 3-fold increase in gallbladder diseases, and 5-fold increase in sleep apnea diagnosed among these children and adolescents over a short period of two years. Abnormal liver enzyme levels have been reported among overweight and obese adolescents, particularly those using alcohol [5]. Additionally, non-alcoholic fatty liver disease has been diagnosed with higher frequency among adolescents [6,7].

In addition to financial and medical consequences, childhood obesity has been documented to be associated with increased risks of low self-esteem [8-10]. This is assumed to be due to the social stigma associated with obesity in Western society [11]. Low self-esteem is a notable consequence of obesity, since those with low self-esteem are less likely to complete a pediatric weight control program [12] and more likely to have unhealthy eating behavior [13]. Low self-esteem in obese children is also linked to the initiation of tobacco and alcohol use [14]. This is significant since adolescent girls often use smoking as a means of weight control [15], leading to recommendations that childhood obesity treatment also include smoking cessation [16]. Alcohol consumption in overweight and obese adolescents significantly increases the risk of abnormal liver enzyme levels [5]. Low self-esteem in obese children has also been shown to be associated with increased levels of loneliness, sadness and nervousness [14]. Those with high self-esteem report greater satisfaction and happiness later in life and better adjustment in school and social relationships [17], and lower levels of psychopathology [18]. Due to the importance of self-esteem in shaping a child’s emotional and physical well-being, it merits study.

There are discrepancies in findings of major studies on self-esteem and obesity in children [19]. The large discrepancies may be due to race/ethnicity, gender, and age differences. Since most studies of self-esteem have used small samples of White, middle-class children, the relationship between body weight and self-esteem among underserved minority children is not well documented. However, this population deserves investigation since obesity disproportionately affects minority children [20], with their weight problems accelerating more rapidly [1], and they are more likely to develop co-morbid psychological maladaptation [21]. Low-income Caucasian women have been shown to make poorer nutritional choices as compared to those from higher socio-economic status [22]. Therefore, low-income minority families may make poorer nutritional choices while their children are being exposed more to media and screen time [23] and are less successful in pediatric weight loss interventions [24]. Additionally, significant racial/ethnic differences exist in weight perception, attempts to lose weight, and weight goals [25]. Many studies have not explicitly researched gender or age differences in examining the relationship between weight and self-esteem, particularly in underserved minority populations. Girls underestimate their body weight while boys overestimate their weight, and there are sex differences in weight loss attempts and weight loss goals in multiethnic youth [25]. Girls also demonstrate a stronger association between weight and self-esteem than do boys [14,26]. There are also gender differences in psychosocial functioning of overweight and obese minority children, with the relation between BMI and body esteem mediated by poor physical health for boys but not girls, and being bullied by peers being associated with lower body esteem only in girls [27]. Age differences are also important in examining body weight and self-esteem. The general self-esteem of overweight children decreases from childhood to early adolescence [28], and older age has been associated with lower levels of physical self-esteem [29]. Mean global self-worth shows little change over ages 9–14 years in blacks but decreases in whites, suggesting that self-esteem may not follow the same developmental pattern in minority groups as in whites [30]. It is clear that the relationship between body weight and self-esteem is complex and mediated by ethnic, gender and age status. This study describes the self-esteem profile in a large sample of underserved minority children living in inner city, low-income neighborhoods in the United States and its association with race/ethnicity, gender, age and body weight.

## Methods

### Study population

A total of 910 minority children aged 9–12 years, regardless of body weight, and living in the Greater Houston Metropolitan area in the United States were enrolled in the Healthy Kids-Houston Study between January 2009 and February 2012. The Healthy Kids-Houston is a community-based after-school program to promote healthy lifestyle among minority children. Each program consists of three 6-week sessions, once in the fall, once in the spring and once at the end of the school year. The children were recruited from economically distressed neighborhoods surrounding 17 community centers managed by the City of Houston Parks and Recreation Department (HPARD) to make sure we had a representative sample of minority children living in the Greater Houston Metropolitan Area. The program was promoted through newsletters of the community centers and at nearby public schools. These schools were populated primarily by minority children, with the majority of them qualifying for free or reduced-cost school meal programs. Data collection was done over a period of three years because we were limited by the physical capacity of the community centers and had to make sure we had sufficient staff to properly implement the program and ensure the safety of the children. All measurements were taken prior to the children receiving any intervention or having significant interactions with the study staff.

The Institutional Review Board for Human Subject Research for Baylor College of Medicine (BCM) and Affiliated Hospitals approved the study protocols. To enroll in the Healthy Kids-Houston study, the parents completed the HPARD enrollment form and the BCM consent form. The program brochures, enrollment forms, and consent forms were available in both English and Spanish.

### Weight and height

Body weight and height were measured in duplicate with an electronic digital scale and with a digital stadiometer, respectively. The body weight of each child without shoes and heavy clothing were measured to the nearest 0.1 kg using a Scale Tronix electronic scale (Model 5600, Scale Tronix Inc, Wheaton IL) which was calibrated with a 25-kg reference weight (Scale Tronix Inc., Wheaton IL) prior to measurement. Height was measured, also without shoes and heavy clothing, with heels, buttock, back, and back of the head touching the stadiometer stand and marked to the nearest 1 mm using an Accustat stadiometer (Model G448, Seca Corp, Columbia, MD). Project staff trained on proper measurement procedures and the proper use of the equipment made the measurements. The average values were used to calculate body mass index (BMI). Children were considered normal-weight if their BMI values were ≥5^th^ percentile but <85^th^ percentile, overweight if their BMI values were ≥85^th^ percentile but <95^th^ percentile, and obese if their BMI values were ≥95^th^ percentile [31].

### Self-esteem profile

To document the self-esteem profile of the study participants, each child completed a Self-Perception Profile for Children (SPPC) [32]. The SPPC is a widely used questionnaire for assessing self-esteem in children [33]. The author views self-esteem as an evaluative aspect of oneself [34]. Individuals experience high self-esteem when there is little discrepancy between the ideal and the perceived real self. Low self-esteem arises when the discrepancy is great. In this 36-item self-report scale, each question provides the child with two descriptions. The child then selects which description is “most like” him or herself, and then rates whether the description is “really true” or “sort of true” for him or herself. There are five specific domains: (1) scholastic competence – the child’s perception of his/her competence or ability in scholastic performance; (2) social acceptance – the degree to which a child perceives they are accepted by peers or feels popular; (3) athletic competence – the child’s perception of his/her competence in sports and outdoor games; (4) physical appearance – the degree to which a child is happy with the way he/she looks; and (5) behavioral conduct – the degree to which the child likes the way he/she behaves and acts the way he/she is supposed to. There is also a general domain of global self-worth or self-esteem. Each domain has six item scores, and is scored either 1 2, 3 or 4 with 4 representing the most adequate self judgement and 1 representing the least adequate self judgement. There are gender and age differences, but in general the standardization sample means fluctuate around a value of 3.0, and the majority of standard deviations fall between .50 and .85. The instrument is designed for children in the third to sixth grade, although it may be used with older subjects. Any self-esteem score below 3.0 is considered low. Bilingual study personnel were readily available to assist the children to complete the questionnaire.

In the standardization sample used for the SPPC manual, the internal consistency of the SPPC scales was satisfactory with Cronbach’s alphas of 0.82 for scholastic competence, 0.78 for social acceptance, 0.83 for athletic competence, 0.72 for physical appearance, 0.74 for behavioral conduct, and 0.80 for global self-worth. The test-retest reliability of the SPPC over a 4-week interval was good with all intraclass correlation coefficients at 0.84 or higher. More specifically, the scale correlated with child-, parent-, and teacher-reports of psychopathology and personality. In factor analysis, the SPPC was shown to consist of five factors that corresponded with the theorized domains of self-esteem [35]. There is support for the use of this instrument with elementary age Mexican American children, where the internal consistency coefficients for the entire instrument was 0.89 and for individual subscales ranged from 0.62 to 0.74 [36]. Concurrent validity was established with moderate correlations with measures of school attitude and academic achievement. In a study evaluating the psychometric properties of the SPPC with African American girls [37], there was moderate internal reliability, with subscale alpha coefficients ranging from 0.71 to 0.82. However, in the same study there was not good evidence of convergent validity with the Rosenberg Self-esteem scale, although the sample consisted of only girls from at-risk environments.

### Socio economic status (SES)

The SES of the children was based on information provided by the parents in the program enrollment form. Children were considered to be from low-income families when they qualified for free or reduced-cost meal program at school and qualified for federal or state medical insurance programs for low-income families.

### Statistical procedures

Descriptive statistics were used to generate the means and standard deviations of all the outcome measures. One-Way ANOVA for continuous variables or Pearson Chi-Square test for categorical variables were used to evaluate differences between the race/ethnic groups. Generalized linear models including all 2- and 3-way interactions were used to test the effects of gender, age (9–10 y *vs.* 11–12 y), race/ethnicity (black *vs*. Hispanic), and obesity status (normal-weight, overweight, obese), while controlling for center (the center from which the children were recruited), on each domain of the SPPC. Non-significant 2- and 3-way interactions, starting with the most non-significant 3-way interaction, were removed from the model one at a time using a backward stepwise elimination procedure. The software program, DIFFER, was used to compare the mean SPPC scores of the minority children in our study with those reported in the literature for normal-weight, overweight and obese white children from Australia [38] and obese children who attended the Kamp K’aana program [39].

## Results

Table 1 describes the demographic and physical characteristics of the 910 minority children, broken down by weight status (normal weight, overweight, obese), enrolled in the Healthy Kids-Houston program. Age and gender distribution did not differ by weight status (P ≥ 0.09). The majority of children (70%) were overweight or obese, with more Hispanic children falling into these categories (77.7% *vs.* 52.2%, P < 0.001) than black. The black children were taller (P < 0.001) but the Hispanic children were heavier by weight (P < 0.02), body mass index (BMI, P < 0.001) and BMI z-score (P < 0.001). No difference in socio-economic status distribution was detected by weight status (P = 0.09). However, more Hispanic children (99.6%) were in the low SES category than black (89.9%, P < 0.001).

**Table 1:** Demographic and physical characteristics of the Healthy Kids-Houston participants by weight status

	**Normal**	**Overweight**	**Obese**	**P**^**b**^
n	272	148	490	
Age, y	10.3 ± 0.9^a^	10.3 ± 0.9	10.3 ± 1.0	0.78
Gender, n (%)				
Male	128 (47.1%)	74 (50.0%)	270 (55.1%)	0.09
Female	144 (52.9%)	74 (50.0%)	220 (44.9%)	
Race/ethnicity, n (%)				
Black	130 (47.8%)	45 (30.4%)	97 (19.8%)	<0.001
Hispanic	142 (52.2%)	103 (69.6%)	393 (80.2%)	
Weight, kg	34.0 ± 6.0	42.8 ± 6.6	59.5 ± 14.4	<0.001
Height, cm	139.2 ± 8.6	141.4 ± 8.6	144.2 ± 8.5	<0.001
BMI, kg/m^2^	17.4 ± 1.6	21.3 ± 1.2	28.3 ± 4.6	<0.001
BMI z-score	0.15 ± 0.62	1.34 ± 0.17	2.20 ± 0.31	<0.001
Socioeconomic status^c^				
High	7.1%	7.6%	3.9%	P = 0.09
Low	92.9%	92.4%	96.1%	

^a^Mean ± SD where applicable.

^b^Significant values by One-Way ANOVA for continuous variables and by Pearson Chi-Square for categorical variables.

^c^Based on qualification for free/reduced cost meals at school and Medicaid.

**Figure 1:**
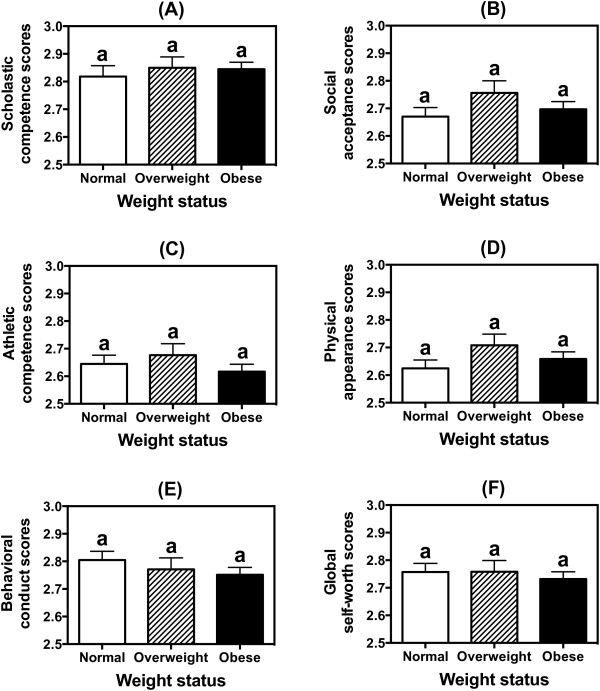
**Effect of body weight on the self-perception profile for children’s domain scores of the minority children. (A)** scholastic competence; **(B)** social acceptance; **(C)** athletic competence; **(D)** physical appearance; **(E)** behavioral conduct; and **(F)** global self-worth. Each domain has six item scores, and is scored either 1, 2, 3 or 4 with 4 representing the most adequate self judgement and 1 representing the least adequate self judgement. Children were considered normal-weight if their BMI values were ≥5^th^ percentile but <85^th^ percentile, overweight if their BMI values were ≥85^th^ percentile but <95^th^ percentile, and obese if their BMI values were ≥95^th^ percentile. Each column represents the estimated mean by generalized linear models after controlling for age, gender, race/ethnicity and centers. The cross bar above each column represents the standard error of the estimated mean. Identical letters above columns indicate no significant difference across weight status.

As shown in Figure 1, weight status was found to have no effect on scholastic competence (Figure 1A, P = 0.71), social acceptance (Figure 1B, P = 0.25), athletic competence (Figure 1C, P = 0.40), physical appearance (Figure 1D, P = 0.22), behavioral conduct (Figure 1E, P = 0.38), and global self-worth (Figure 1F, P = 0.73) after adjusting for age, gender, race/ethnicity and centers. The figure also shows that with the exception of scholastic competence scores (Figure 1A) and the behavioral conduct scores among the normal-weight children (Figure 1E), all the other scores were below the 2.80 unit. More importantly, only behavioral conduct (Figure 1E) showed a potential downward trend with increasing body weight.

Table 2 summarizes the Self-Perception Profile for Children’s domain scores by race/ethnicity. After adjusting for age, gender, weight status, and center, black children had higher scholastic competence (black: 2.88 ± 0.03, mean ± SE *vs.* Hispanic: 2.79 ± 0.03, P = 0.05) than Hispanic children. Social acceptance was not affected by age (P = 0.69), gender (P = 0.13), race/ethnicity (P = 0.32) or weight status (P = 0.25).

**Table 2:** Estimated means for Self-Perception Profile for Children’s domain scores among the minority children

	**Black**	**Hispanic**	**P**
n	272	638	
Scholastic competence	2.88 ± 0.03^a^	2.79 ± 0.03	0.05
Social acceptance	2.89 ± 0.05	2.82 ± 0.04	0.32
Athletic competence	2.66 ± 0.04	2.63 ± 0.03	0.50
Physical appearance	2.71 ± 0.04	2.62 ± 0.03	0.05
Behavioral conduct	2.82 ± 0.04	2.74 ± 0.03	0.11
Global self-worth	2.74 ± 0.04	2.76 ± 0.03	0.71

^a^Estimated mean ± SE by generalized linear model with adjustment for age, gender, weight status and community centers.

Significant age x gender (P = 0.006) and race x gender (P = 0.005) interactions were detected on athletic competence scores. The younger boys (9–10 y) had higher athletic competence scores (2.72 ± 0.03) than the younger girls (2.62 ± 0.03, P = 0.01) and the older girls (2.53 ± 0.05, P = 0.001). The older boys (11–12 y) also had higher athletic competence scores (2.71 ± 0.05) than the older girls (P = 0.008), but their scores were not statistically different from those of the younger girls (P = 0.07). Within each race/ethnic group, boys had higher athletic competence scores than girls (2.72 ± 0.03 *vs.* 2.57 ± 0.03, P = 0.001). Black boys had higher athletic competence scores (2.73 ± 0.05) than Hispanic girls (2.55 ± 0.04, P = 0.007) but their scores were not statistically different from those of the Hispanic boys (2.71 ± 0.04, P = 0.69). Athletic competence scores were not different between the black girls and the Hispanic girls (P = 0.51).

Physical appearance scores were not affected by age (9–10 y: 2.67 ± 0.02 *vs.* 11–12 y: 2.65 ± 0.03, P = 0.48) or gender (boys: 2.68 ± 0.03 *vs*. girls: 2.64 ± 0.03, P = 0.20). However, black children had higher scores (2.71 ± 0.03) than the Hispanic children (2.62 ± 0.03, P = 0.05).

Behavioral conduct scores were not related to age (9–10 y: 2.81 ± 0.02 *vs*. 11–12 y: 2.74 ± 0.03, P = 0.09), gender (boys: 2.79 ± 0.03 *vs*. girls: 2.76 ± 0.03, P = 0.34), and race/ethnicity (black: 2.81 ± 0.03 *vs*. Hispanic: 2.74 ± 0.03, P = 0.11).

There was an age x gender interaction on global self-worth scores (P = 0.02). Both the younger and older boys had higher scores (9–10 y: 2.82 ± 0.03 and 11–12 y: 2.78 ± 0.04) than the older girls (2.65 ± 0.05, P ≤ 0.04), but their scores were not statistically different from those of the younger girls (2.75 ± 0.04, P ≥ 0.06).

## Discussion

Our results showed that the self-esteem profile scores are similar among the minority children regardless of their weight status (Figure 1). Therefore, being normal weight did not necessarily guarantee positive self-esteem in our sample, and being overweight or obese did not necessarily mean that participants had a lower self-esteem. It may be that being a member of a minority group plays a more salient role in determining one’s self-esteem than does body weight. We compared the scores of the Healthy Kids-Houston minority children with the SPPC scores of children diagnosed with obesity and obesity-related co-morbidities [39]. Importantly, but sadly, with the exception of athletic competence and physical appearance, the scores for scholastic competence (Healthy Kids: 2.83 ± 0.45 *vs*. obese kids: 2.74 ± 0.52, P = 0.08), social acceptance (Healthy Kids: 2.69 ± 0.51 *vs*. obese kids: 2.69 ± 0.54, P = 1.00), behavioral conduct (Healthy Kids: 2.77 ± 0.49 *vs*. obese kids: 2.77 ± 0.52, P = 1.00) and global self-worth (Healthy Kids: 2.74 ± 0.48 *vs.* obese kids: 2.71 ± 0.52, P = 0.56) among both groups were statistically similar.

Our results also showed that black children have better self-esteem in terms of scholastic competence and physical appearance than the Hispanic children. We speculate that the lower scores among the Hispanic children could possibly be related to their lack of integration into American society, language barrier, or that they perceive themselves as belonging to a subordinate group to a greater extent. Blacks may have a more positive self-identity which emphasizes their desirable distinctiveness or may have more psychological resources that enable them to deflect the negative perceptions [40]. Black respondents have been shown to evaluate in-group characteristics more positively than out-group characteristics [41], and the self-esteem of black youth has been shown to be more internally motivated and less contingent on external motivators such as other’s approval or physical appearance [42]. Body satisfaction has been more frequently examined in black girls than boys. Studies show that black girls may experience less body dissatisfaction due to their preference for a larger ideal body size [43]. This is consistent with results that black girls equate large body types with strong female figures present in their life so that size corresponds to strength [44]. Alternatively, they may be using a number of factors other than size to determine athletic prowess. Further investigation is needed to determine mediators of high self-esteem in black boys. Since the older girls had the lowest self-esteem scores when compared to the younger girls and the boys, programs to promote self-esteem among minority children should start at young ages, particularly among girls. Additionally, the low scores of Hispanic children on scholastic competence and physical appearance suggest the value in providing programs targeting self-esteem specifically for this vulnerable population.

When compared to Australian white children [38], our results showed that underserved minority children, regardless of their body weight, have significantly lower self-esteem than normal-weight white children (e.g. global self-worth: minority children, 2.78 ± 0.49 *vs.* white children, 3.28 ± 0.59) and in many cases, lower than those reported for overweight (3.07 ± 0.63) and obese white children (3.00 ± 0.70). A meta-analysis of studies measuring academic and global self-esteem in subjects younger than 23 years old [40] showed young black children had lower self-esteem than white children, but this gap diminished or even reversed through adolescence. Another meta-analysis [45] found the self-esteem of black children to be highest, followed by that for white and then Hispanic children. This effect was very slight in elementary age children, and the gap widened with age, peaking at college age. The fact that in our study older minority girls had lower global self-worth scores suggests that they may have had more time to internalize negative stereotypical attitudes and stigma. Further research is necessary to determine if this finding applies only to a Southern US city or is a more pervasive problem.

The white children from Australia, because of different geographical location and environmental conditions, might not be the ideal reference population [38]. However, the publication provided the largest set of SPPC scores broken down by weight and by gender. We are not aware of any other publications in the United States that broke down the SPPC scores by weight and by gender. Furthermore, based on the data that we have collected from the obese children (22 black, 48 Hispanic, 46 white) who attended the Kamp K’aana program [39], only two scores (athletic competence and physical appearance) were higher among the minority children in the Healthy Kids-Houston program when compared to the Kamp K’aana obese children suggesting that low self-esteem scores might be common among minority children regardless of race/ethnicity and gender.

Another limitation of this study is the fact that self-report may not always accurately reflect the construct of self-esteem, particularly in minority groups who may have different response styles or self-presentation. However, the SPPC is a highly validated instrument to document self-esteem among children and has been shown to be appropriate for Hispanic and black children [33,35-37].

The lack of a control group, either a minority group or a group of white children, is a limitation of the current study. However, children from affluent families probably are not living in the same distressed neighborhoods of our minority children. Therefore, these children would not be an ideal control group for our underserved minority children. Another limitation is the cross-sectional nature of the data collection.

A large number of statistical tests are done giving rise to multiple comparison issues. However, since results for the primary question regarding the effect of BMI on self-esteem was not significant, any adjustment would only make it more non-significant and there would be no substantive change to the conclusions. The findings regarding demographic variables such as age, gender, and race/ethnicity are secondary and should be considered as preliminary.

## Conclusions

Self-esteem appears to be low and moderated by ethnicity, gender and age, but not by weight status, in underserved minority children. However, there was also some intra-group variation suggesting that some children were able to maintain a better self-esteem in spite of group membership. Further research could elaborate on the specific mechanisms that contribute to these differences in order to assist minority children in improving self-esteem. Since low self-esteem has been shown to relate to unhealthy eating behavior [13] and the initiation of tobacco and alcohol use [46-49], programs to improve self-esteem among minority children living in inner city, low-income neighborhoods are needed in order to help them achieve a healthy lifestyle and to prevent them from acquiring the co-morbidities related to obesity.

## Abbreviations

BCM: Baylor College of Medicine; BMI: Body mass index; HPARD: City of Houston Parks and Recreation Department; SPPC: Self-Perception Profile for Children.

## Competing interests

The authors declared that they have no competing interests. The contents of this publication do not necessarily reflect the views or policies of the U.S. Department of Agriculture or mention of trade names, commercial products, or organizations imply endorsement.

## Authors’ contributions

WWW was responsible for the implementation of the Healthy Kids-Houston that generated the self-esteem data for the manuscript. CM trained the staff on the collection of self-esteem data using the SPPC instrument. CLO, DL and LAM identified the community centers to support the project. They also provided the instructors to help collect the data. KLK assisted in subject recruitment and staff training. EOS assisted in the study design and the statistical analysis of the data. All authors were involved in the original study design, assisted in the implementation of the project, and read and approved the final manuscript.

## Authors’ information

WWW is the Project Director of the Healthy Kids-Houston project and a Professor of Pediatrics at BCM. CM is a Co-Investigator of the Healthy Kids-Houston project, a Clinical Psychologist at Texas Children’s Hospital, and an Associate Professor of Pediatrics at BCM. CLO is the Principal Investigator of the Healthy Kids-Houston project and an Administrator Manager at HPARD. DL is the Assistant Director at HPARD. LAM is a Co-Investigator of the Healthy Kids-Houston project and a Senior Superintendent at HPARD. KLK is a Project Consultant of the Healthy Kids-Houston project. EOS is the Biostatistician of the Healthy Kids-Houston project and a Professor of Pediatrics at BCM.

## Pre-publication history

The pre-publication history for this paper can be accessed here:

http://www.biomedcentral.com/1471-2431/14/19/prepub
